# Cannabis Affects Cerebellar Volume and Sleep Differently in Men and Women

**DOI:** 10.3389/fpsyt.2021.643193

**Published:** 2021-05-13

**Authors:** Katherine L. McPherson, Dardo G. Tomasi, Gene-Jack Wang, Peter Manza, Nora D. Volkow

**Affiliations:** ^1^National Institute on Alcohol Abuse and Alcoholism, National Institutes of Health, Bethesda, MD, United States; ^2^National Institute on Drug Abuse, National Institutes of Health, Bethesda, MD, United States

**Keywords:** marijuana, tetrahydrocannabinol, magnetic resonance imaging, sexual dimorphism, subcortical volume

## Abstract

**Background:** There are known sex differences in behavioral and clinical outcomes associated with drugs of abuse, including cannabis. However, little is known about how chronic cannabis use and sex interact to affect brain structure, particularly in regions with high cannabinoid receptor expression, such as the cerebellum, amygdala, and hippocampus. Based on behavioral data suggesting that females may be particularly vulnerable to the effects of chronic cannabis use, we hypothesized lower volumes in these regions in female cannabis users. We also hypothesized poorer sleep quality among female cannabis users, given recent findings highlighting the importance of sleep for many outcomes related to cannabis use disorder.

**Methods:** Using data from the Human Connectome Project, we examined 170 chronic cannabis users (>100 lifetime uses and/or a lifetime diagnosis of cannabis dependence) and 170 controls that we attempted to match on age, sex, BMI, race, tobacco use, and alcohol use. We performed group-by-sex ANOVAs, testing for an interaction in subcortical volumes, and in self-reported sleep quality (Pittsburgh Sleep Questionnaire Inventory).

**Results:** After controlling for total intracranial volume and past/current tobacco usage, we found that cannabis users relative to controls had smaller cerebellum volume and poorer sleep quality, and these effects were driven by the female cannabis users (i.e., a group-by-sex interaction). Among cannabis users, there was an age of first use-by-sex interaction in sleep quality, such that females with earlier age of first cannabis use tended to have more self-reported sleep issues, whereas this trend was not present among male cannabis users. The amygdala volume was smaller in cannabis users than in non-users but the group by sex interaction was not significant.

**Conclusions:** These data corroborate prior findings that females may be more sensitive to the neural and behavioral effects of chronic cannabis use than males. Further work is needed to determine if reduced cerebellar and amygdala volumes contribute to sleep impairments in cannabis users.

## Introduction

There are marked sex differences in the acute and long-term effects of drugs of abuse, including subjective effects, neurological impact, and behavioral outcomes. These disparate effects may be due to differences in metabolism, body fat and water distribution, hormones, and sexual dimorphism in brain function. For example, differences in metabolism and bioavailability cause higher blood alcohol levels in females and greater vulnerability to the negative effects of alcohol, compared to males consuming the same amount of alcohol (1, 2). Greater drug effects in females are thought to contribute to “telescoping,” the observation that women tend to progress from first use to seeking treatment for cannabis use disorder (CUD) more rapidly than men. This phenomenon has been described across several drugs of abuse, including cannabis use disorders (CUD) (3). Despite this, the prevalence of cannabis use and CUD is higher in males than females (4), which is driven by a greater rate of drug initiation among men than women, though this gap is narrowing (5). Along with this acceleration to CUD, women also experience stronger cannabis withdrawal symptoms than men during periods of abstinence (5), as well as worse outcomes on experimental cannabis therapies such as buspirone (6) and vilazodone (7).

These differences in responses to cannabis are likely related in part to sex differences in the function and structure of subcortical brain regions rich in cannabinoid-type I receptors (CB1-R, the primary receptor target for THC, the main psychoactive component of cannabis), such as the cerebellum, amygdala, and hippocampus (8). For instance, rats repeatedly treated with THC exhibited CB1-R desensitization and downregulation in cerebellum, hippocampus, prefrontal cortex, and striatum, with greater effects in females consistent with the “telescoping” observation (9) and which may be dependent on the estrous cycle (10). Chronic THC treatment also had lasting effects in primates, with THC concentration in the cerebellum approximately double the concentration in blood 24 h after the last dose of THC, indicating that brain regions with high CB1R density can be impacted long after cannabis use (11). Importantly, in individuals with CUD the cerebellum showed significant reductions in brain glucose metabolism during withdrawal whereas its activation during cannabis intoxication was associated with its reinforcing effects (12). Moreover, it has been proposed that the effects of cannabis on the cerebellum are relevant to cannabis addiction (13). As it relates to sex differences brain imaging studies showed that in individuals with CUD, females compared to males showed a blunted metabolic response to a stimulant challenge, which was most prominent in CB1-R-dense regions: cerebellum, hippocampus, and thalamus (14). Sex differences in the brain and behavior of cannabis users may also be critically related to sex differences in sleep quality, which is recognized as a factor impacting long-term outcomes in people with CUD (15). However, very little work has been done to describe the possible neurobiological underpinnings of sex differences in humans with a history of chronic cannabis use.

A broad literature has been devoted to understanding the effects of cannabis use on subcortical brain volumes. Findings have been inconsistent, with some studies finding substantially smaller subcortical volumes in chronic cannabis users compared to controls (16–18), whereas others have reported that after controlling for key confounding variables like tobacco usage, these differences are virtually non-existent (19, 20). We and others have argued that these discrepant findings are due to generally small sample sizes and inadequate matching on control groups (21). Nevertheless, several recent reviews have been devoted to the topic (22–24) and some consensus seems to have emerged that cerebellum, amygdala, and hippocampus volumes appear to be most consistently affected by chronic cannabis use across studies (8). However, whether these differences are moderated by sex, and are associated with behavioral outcomes such as sleep quality remains unknown.

Current findings regarding cannabis use and sleep quality are mixed, particularly when considering sex differences. Previous studies using the Pittsburgh Sleep Quality Index self-report scale (PSQI) among generally healthy adults, reported that women had lower scores on sleep quality (25–28), sleep efficiency (27), and higher sleep disturbances (28) than men, suggesting that sex differences in sleep quality may exist even before taking substance use into account. Chronic cannabis use can further complicate this picture. Acute withdrawal from cannabis can contribute to objective and subjective sleep disturbances, which are more common in chronic users (29, 30). Acutely cannabis can decrease sleep latency, making it easier to fall asleep (31, 32); however, long-term sleep quality is negatively impacted (15). In fact, roughly half of adults with CUD reported that cannabis use had caused them difficulty sleeping in the past 90 days (33). Heavy users also reported a decrease in desirable sleep aftereffects (e.g., restful sleep, duration) over time (34). Females compared to males who had “risky” use of both alcohol and cannabis reported especially poor sleep quality reflected by high PSQI total scores (35), but it was not clear whether alcohol or cannabis use was most associated with this pattern. In sum, while cannabis use and sex can have strong effects on sleep quality, we are not aware of any studies that have investigated the interaction between these two factors. This is particularly relevant given a wide body of work that chronic impaired sleep quality can negatively impact brain structure [e.g., (36)].

Together, converging evidence suggests that there are sex differences in the effects of chronic cannabis use on subcortical brain volumes and sleep. However, the interaction of sex on cannabis effects on subcortical brain volumes and sleep quality has not been investigated. To address this neglect, we took advantage of Human Connectome Project data (37) to examine brain structure and sleep quality in a relatively large number of participants with a history of chronic cannabis use and well-matched controls. We hypothesized that female cannabis users would have smaller volumes in amygdala, hippocampus, and cerebellum, which are subcortical regions dense with CB1-Rs (38), and poorer sleep quality than male cannabis users.

## Materials and Methods

### Participants

Participants included in this study provided written informed consent at Washington University in St. Louis (39). Out of 1,005 individuals with structural MRI data in the Human Connectome Project, we identified 170 individuals meeting DSM-IV criteria for lifetime (current or prior) CUD and/or >100 lifetime cannabis uses, and without comorbid current or prior alcohol dependence, as in our previous work (21, 40), which became the cannabis group (CAN). We also selected a control group (CTL; *n* = 170) with <10 lifetime cannabis uses, and used the matchControls package in R to try and match controls with the CAN group on: age, sex, education, BMI, race, and a composite measure reflecting past/current alcohol usage (41, 42). Of note, we could not match on tobacco usage, which was higher in the CAN group (*p* < 0.001), and subsequent analyses were performed to ensure results were not driven by past/current tobacco usage. For more details on participant demographics see Table 1.

**Table 1:** Demographics and clinical characteristics for chronic cannabis users (CAN) and controls (CTL).

	**Mean (SD)**	**Mean (SD)**	**M vs. F: *T-stat, p***	**CAN vs. CTL: *T-stat, p***
**Cannabis (CAN)**	**Males (*****n*** **=** **114)**	**Females (*****n*** **=** **56)**		
Age	27.614 (3.635)	28.714 (3.944)	−1.754, 0.082	−0.247, 0.805
BMI	26.033 (4.110)	27.477 (6.320)	−1.556, 0.124	−0.284, 0.777
Edu	14.465 (1.825)	14.018 (1.995)	1.411, 0.161	−2.039, 0.042
Tobacco use (Composite-Z)	0.647 (1.082)	0.366 (1.096)	1.578, 0.118	3.246, 0.001
Alcohol use (Composite-Z)	0.218 (0.424)	0.061 (0.341)	2.600, 0.010	0.901, 0.368
% Caucasian	72.81	57.14	χ^2^ = 4.210, *p* = 0.040	
% Black/African American	15.79	30.36	χ^2^ = 4.874, *p* = 0.027	
**Controls (CTL)**	**Males (*****n*** **=** **114)**	**Females (*****n*** **=** **56)**		
Age	27.658 (3.604)	28.929 (3.756)	−2.101, 0.038	
BMI	26.406 (3.953)	27.163 (5.048)	−0.976, 0.332	
Edu	14.702 (1.755)	14.768 (1.849)	−0.223, 0.824	
Tobacco use (Composite-Z)	0.189 (0.987)	0.191 (0.976)	−0.015, 0.988	
Alcohol use (Composite-Z)	0.184 (0.376)	0.018 (0.249)	3.431, 0.001	
% Caucasian	74.56	58.93	χ^2^ = 4.323, *p* = 0.038	
% Black/African American	15.79	30.36	χ^2^ = 4.874, *p* = 0.027	

### MRI Image Acquisition and Preprocessing

Scans were collected using a custom-made Siemens Connectom Skyra scanner with a 32-channel head coil. T1- and T2-weighted anatomical scans were acquired at 0.7 mm isotropic resolution (37). Structural images were “minimally preprocessed” by HCP investigators through standardized pipelines (43). Images were corrected for gradient non-linearity-induced distortions, readout distortions, and intensity inhomogeneities, and then aligned to the MNI atlas. Then, images were processed through a customized version of Freesurfer. We used the volume values for all subcortical regions (averaged across the left and right regions, where possible) in the Desikan-Killany parcellation (44), which resulted in the analysis of 10 regions: Amygdala, Hippocampus, Putamen, Caudate, Nucleus Accumbens, Thalamus, Pallidum, Brainstem, Cerebellar Cortex, and Cerebellar White Matter.

### Self-Reported Sleep Quality

The Pittsburgh Sleep Quality Index (PSQI) was developed in 1988 to assess sleep via 19 questions; it produces a validated global score based on seven sub-scores such as efficiency, quality, and disturbances (45).

### Statistical Analyses

Analyses were performed in R version 3.6.2 and in GraphPad Prism version 8.0.1. To test for sex differences in subcortical regional volumes, we constructed linear regression models using the lm Function in R (equivalent to an analysis of variance), where the main effects of sex and cannabis group membership (and their interaction) were the predictor variables, tobacco usage and total intracranial volume were covariates, and each region's subcortical volume was the outcome variable. To correct for multiple comparisons across all 10 regions of interest, we used false discovery rate (Benjamini-Hochberg) correction. We also tested for differences in self-reported sleep quality using the same analytical approach, except that total PSQI score was the outcome variable.

To attempt to link any of the above findings that showed significant cannabis group-by-sex interactions, we performed mediation analysis. We tested whether sleep scores mediated the association between sex and subcortical volumes, using the causal mediation analysis toolbox in R (46) with 1,000 permutations. We also tested the reverse mediation analysis: that subcortical volumes mediated the association between sex and self-reported sleep quality. In these analyses we used only the data from participants in the CAN group (*n* = 170), and we controlled for tobacco usage and total intracranial volume.

Finally, we tested if any of the subcortical volumes or self-reported sleep quality with significant cannabis group-by-sex interactions were driven by participants who had an earlier age of cannabis use onset, since this has been associated with poorer outcomes in cannabis users generally, and in our prior study with differences in subcortical function (42). The HCP recorded age of first cannabis use on an ordinal scale (1: <14 years old, 2: 15–17 years old, 3: 18–20 years old, 4: 21+ years old). We therefore tested for interaction effects by performing sex-by-age of first use ANOVAs, using only the data from participants in the cannabis use group (*n* = 170), again controlling for tobacco usage and total intracranial volume.

## Results

**Figure 1:**
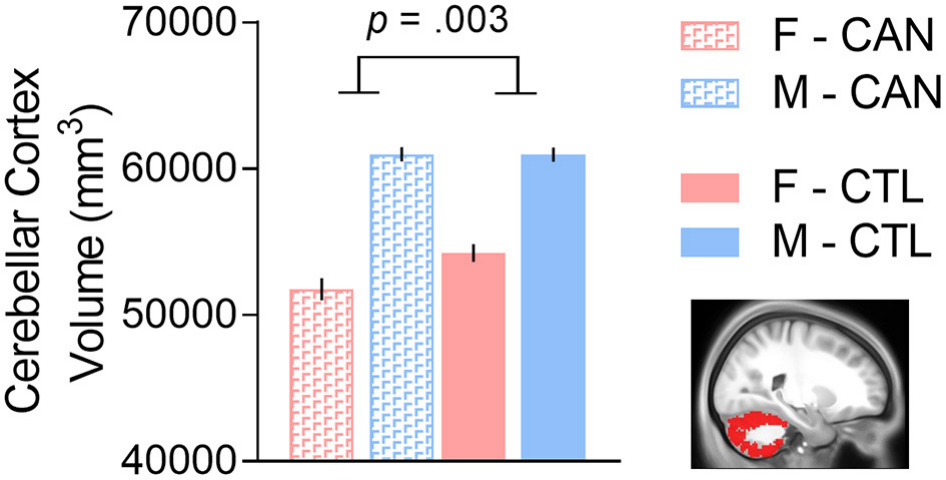
There was a cannabis group-by-sex interaction in cerebellar cortex volume, such that females with a history of chronic cannabis use had smaller volumes than the other groups. We controlled for tobacco usage and total intracranial volume in the analysis. F, Female; M, Male; CAN, Chronic cannabis use group; CTL, control group.

We constructed linear models to determine if the interaction of sex and chronic cannabis usage was significantly associated with subcortical volumes, controlling for tobacco usage and total intracranial volume. We first noted that there were no significant main effects of sex in any of the 10 regions tested after FDR correction (all *p*'s > 0.20). There was a main effect of group in the cerebellar cortex [*t*_(1,334)_ = −3.353, FDR-corrected *p* = 0.008], which was driven by the female cannabis users having lower cerebellar volumes than the other participants [interaction effect: *t*_(1,334)_ = −3.699, FDR-corrected *p* = 0.002; Figure 1]. There was also a trend for a main effect of group in the amygdala [*t*_(1,334)_ = −2.611, FDR-corrected *p* = 0.047], with CAN having lower amygdala volumes than controls, but the sex interaction effect was not significant. No other region (including amygdala) showed a significant group or interaction effect (all *p*'s > 0.35; for full results, see Table 2).

**Table 2:** Summary statistics for analysis of subcortical regional volumes, showing: (1) the main effects of group, i.e., the chronic cannabis use group (CAN) vs. controls (CTL); (2) the main effect of sex, i.e., Males (M) vs. Females (F); and (3) their interaction.

**Region**	**Group: CAN > CTL *t* (*p_***adj***_*)**	**Sex: M > F *t* (*p_***adj***_*)**	**Interaction: *t* (*p_***adj***_*)**
Cerebellar cortex	**−3.353 (0.009)**	1.82 (0.232)	**3.699 (0.003)**
Cerebellar WM	−0.835 (0.763)	1.126 (0.473)	1.323 (0.374)
Amygdala	**−2.611 (0.047)**	1.884 (0.232)	1.78 (0.374)
Hippocampus	−1.341 (0.603)	1.228 (0.473)	1.434 (0.374)
Putamen	0.623 (0.763)	1.85 (0.232)	−0.1 (0.921)
Caudate	−0.422 (0.842)	−0.974 (0.473)	0.413 (0.756)
Accumbens	−1.101 (0.679)	1.059 (0.473)	1.446 (0.374)
Thalamus	−0.266 (0.871)	0.379 (0.881)	0.681 (0.633)
Pallidum	0.731 (0.763)	−0.07 (0.998)	0.665 (0.633)
Brainstem	0.162 (0.871)	−0.003 (0.998)	0.911 (0.605)

*All p-values are adjusted using False Discovery Rate Benjamini-Hochberg Correction. WM, White Matter. Bold values denote statistical significance (p < 0.05)*.

**Figure 2:**
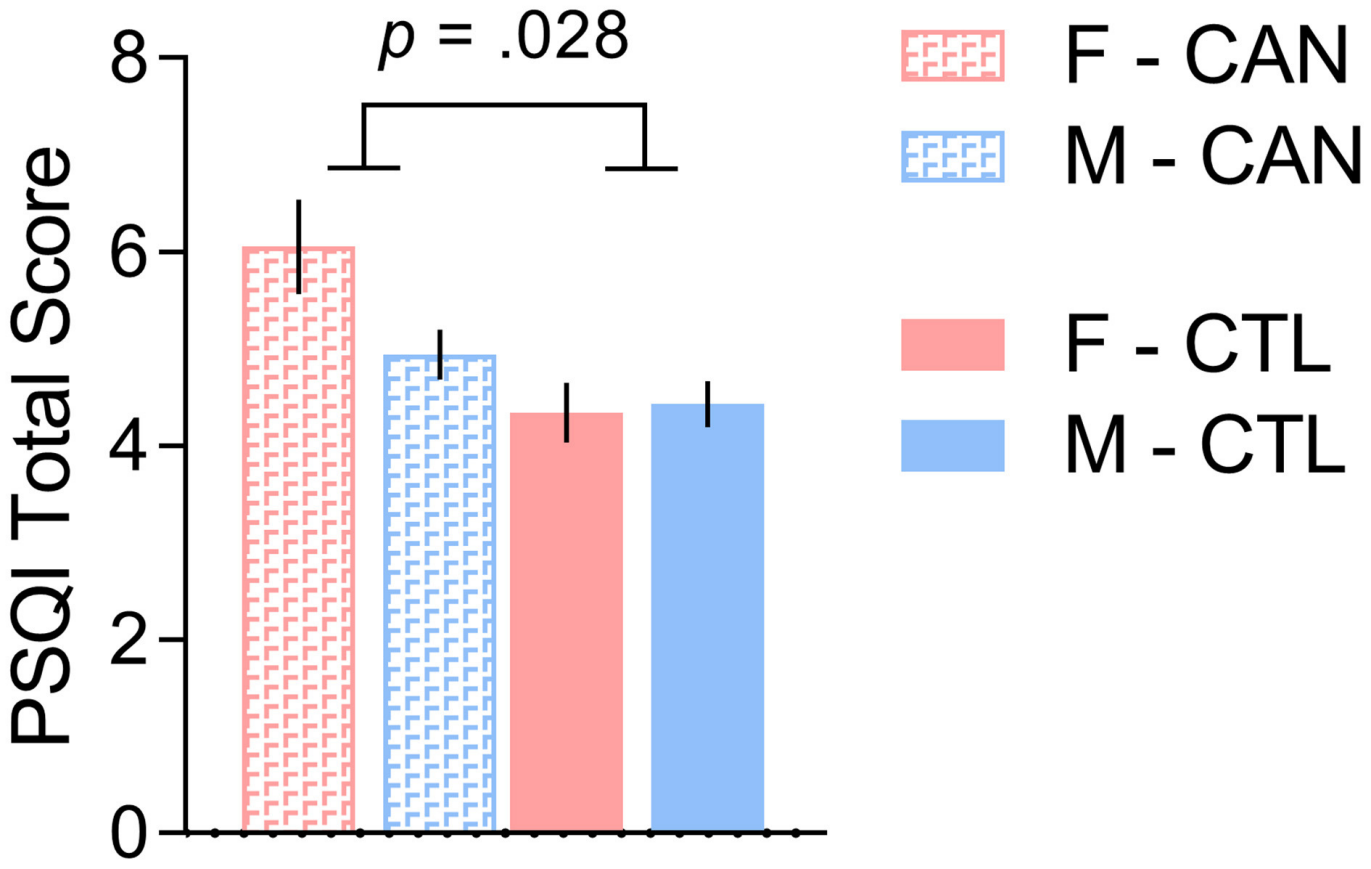
There was a cannabis group-by-sex interaction in self-reported sleep quality, such that females with a history of chronic cannabis use reported more sleep problems than the other groups. We controlled for tobacco usage and total intracranial volume in the analysis. F, Female; M, Male; CAN, Chronic cannabis use group; CTL, control group; PSQI, Pittsburgh Sleep Quality Index.

We further tested whether the interaction of sex and chronic cannabis usage was associated with self-reported sleep quality, again controlling for tobacco usage and total intracranial volume. There was no significant main effect of sex [*t*_(1,334)_ = 1.323, *p* = 0.187], however there was a main group effect [*t*_(1,334)_ = 3.233, *p* = 0.001], which was also driven by the female cannabis users having poorer sleep quality than the other participants [interaction effect: *t*_(1,334)_ = −2.208, *p* = 0.028; Figure 2]. In exploratory analysis, we tested whether cerebellum volume was correlated with self-reported sleep quality among the female cannabis users only, but did not observe a significant effect: *t*_1,52_ = 0.418, *p* = 0.677.

However, there were no significant mediation effects in the models we tested. Among the cannabis group only (*n* = 170), sleep scores (total PSQI score) did not significantly mediate the sex differences in cerebellar volume: [mediation effect estimate = 15.20, 95% CI = (−208.0, 202.6), *p* = 0.93; direct effect estimate = 4,950, 95% CI = (3,030, 6,890), *p* < 1 × 10^−16^]. Likewise, in the reverse model, cerebellar volumes did not significantly mediate the sex differences in sleep scores: [mediation effect estimate = −0.064, 95% CI = (−0.607, 0.470), *p* = 0.82; direct effect estimate = −0.510, 95% CI = (−2.045, 0.980), *p* = 0.58].

**Figure 3:**
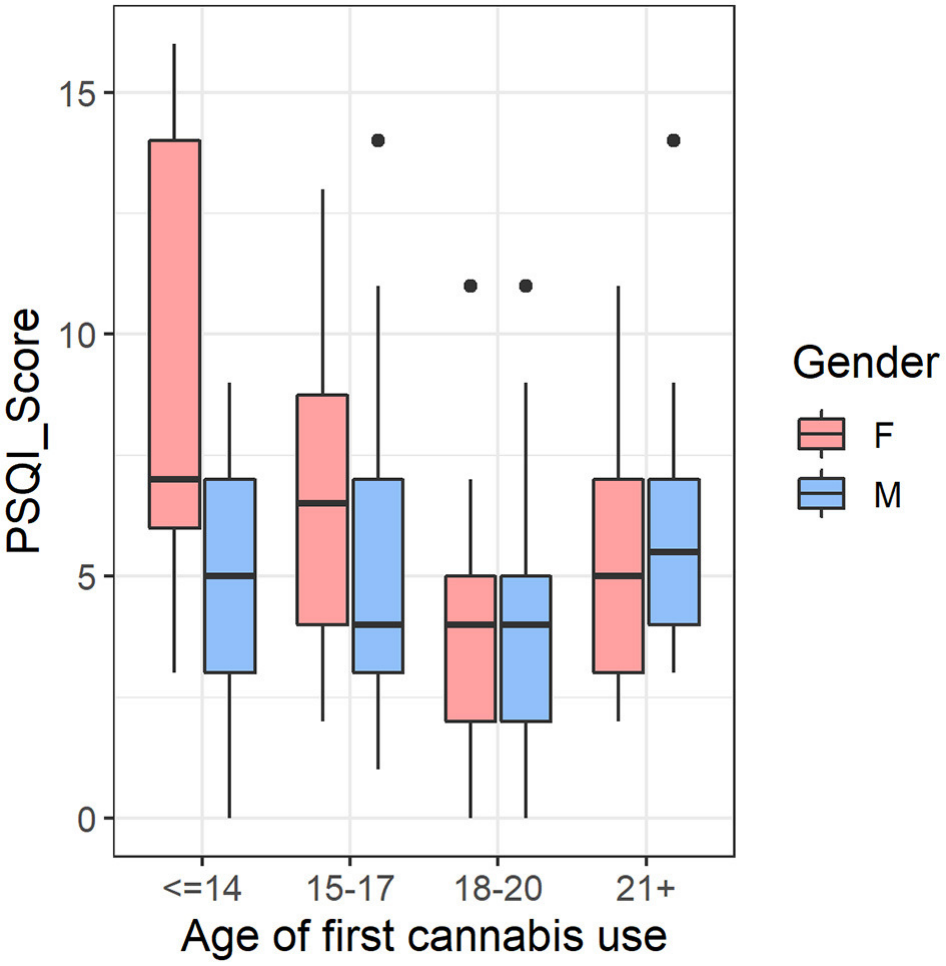
In the cannabis group (CAN) (*n* = 170), there was an age of first use-by-sex interaction in self-reported sleep quality, such that females with earlier age of first cannabis use tended to have more self-reported sleep issues, whereas this trend was not present in male cannabis users. We controlled for tobacco usage and total intracranial volume in the analysis. F, Female; M, Male; PSQI, Pittsburgh Sleep Quality Index.

Finally, we tested if the significant interaction results in cerebellum volume and sleep were driven by female participants who had an earlier age of cannabis use onset in the CAN group (*n* = 170). The cerebellar volumes showed significant main effects of sex [*F*_(1,160)_ = 168.764, *p* < 1 × 10^−16^] and age of first cannabis use [*F*_(3,160)_ = 3.812, *p* = 0.011] but their interaction was not significant [*F*_(3,160)_ = 1.583, *p* = 0.196]. However, for total PSQI score, we observed significant main effects of sex [*F*_(1,160)_ = 5.179, *p* = 0.024], age of first use [*F*_(3,160)_ = 4.077, *p* = 0.008], and their interaction, [*F*_(3,160)_ = 3.587, *p* = 0.015], such that females with earlier age of first cannabis use tended to have more self-reported sleep issues, whereas this trend was not present in male cannabis users (Figure 3).

## Discussion

Our investigation of the impact of cannabis abuse on various subcortical regions yielded results that add to a body of recent work using Human Connectome Project (HCP) data. For instance, recent cannabis use in this sample was negatively associated with hippocampal volume (47) and smaller left hippocampal volume mediated the association between frequency of cannabis use and working memory deficits in cannabis users (48). Additionally, HCP data has revealed an effect of THC exposure on amygdala microstructure organization (49). In line with these studies, we found that cannabis users had marginally smaller amygdala volumes than non-users. However, we only found a cannabis use-by-sex interaction in cerebellar cortex volumes, suggesting that females may be particularly susceptible to the effects of chronic cannabis use in this region. Finally, we observed that female cannabis users had poorer self-reported sleep quality than the other groups, which was particularly pronounced among females who began using cannabis in early adolescence. We discuss these findings in more detail below.

The cerebellum has traditionally been studied for its role in balance and motor coordination (50), nociception (51), and motor cognition (52, 53). Brain imaging studies in humans have shown that the cerebellum is sensitive to the acute and chronic effects of cannabis (8), including glucose metabolic activity (12, 54), volume, and resting-state activity (13, 55–57). Postmortem studies have found striking differences in the cerebellar structure of drug abusers relative to controls; one group showed increased autophagy biomarkers in the cerebellum of multi-substance drug abusers (58), while another found signs of neurodegeneration in the cerebellar cortex of people who were dependent on opioids, suggesting that drug addiction can negatively impact cerebellar structure (59). Recently, Gil-Miravet et al. found that the cerebellum modulates drug-cue associative memory in cocaine users (60), while Hung et al. showed increased functional connectivity between the pallidum and cerebellum of ketamine users, suggesting that the cerebellum has a fundamental role in the pathophysiology of addiction (61). The cerebellum is clearly affected by cannabis use as well; chronic cannabis users can experience cerebellar-dependent motor adaptation impairment (62), while synthetic cannabinoid users show reduced gray matter volume in the left cerebellum (63). These studies are consistent with several recent reviews published on the topic which note the cerebellum's role as a nexus between motor, reward, and cognitive processes crucial to drug seeking behavior (64–66). Compared to other brain regions, there is a relatively high concentration of CB1-Rs in the cerebellum (38, 67, 68). PET studies have shown that CB-1Rs are reversibly downregulated in people with a history of chronic cannabis consumption, which is likely to contribute to tolerance and dependence with repeated use (69). Previous studies have had mixed findings on the relationship between chronic cannabis use and cerebellar volumes with some studies suggesting that cannabis actually increases gray matter volumes (57, 70–73). Here we found that the smaller cerebellar cortical volumes in cannabis users relative to the controls were driven by the female cannabis users. This could explain the discrepancies in the literature since sex was not accounted for in prior investigations. Indeed, studies finding larger cerebellar volumes in cannabis users had very few or no female participants in the cannabis group: Wang et al.: 25% female (5F/15M); Cousijn et al.: 36% Female (12F/21M); Battistella et al.: 0% Female (0F/31M); Wu and Yang: 25% female (5F/15M); Koenders et al.: 25% female (5F/15M). Those findings contrast with the results in our 33% Female sample (56F/114M) and those of another related study (50% Female; 13F/13M) that found lower cerebellar microstructural integrity in adults at risk for CUD relative to controls (56). These findings underscore the importance of including an adequate number of female participants and of investigating sex differences in brain and behavioral outcomes for people with chronic substance use, for such differences appear to be prevalent throughout the addiction endophenotype (8, 74, 75).

We also observed a trend for a group effect on amygdala volume, with lower volumes in cannabis users compared to healthy controls, in agreement with prior studies (73, 76), and which correlated with amount of cannabis used and dependence severity (70, 73). In terms of sex differences, one study found that while adolescent female cannabis users had larger right amygdalar volumes than healthy controls, there was no such difference in males (77). However, our finding of lower amygdalar volume in cannabis users was not sex-dependent and likely would not contribute to the sex-dependent impairment in sleep quality that we observed. Nonetheless, it is possible that a deficit in amygdala volume could contribute to the overall poorer sleep quality observed in CUD compared to controls. The amygdala has been previously implicated in poor sleep quality; while functional connectivity between the amygdala and premotor cortex is negatively associated with sleep quality (78), it appears that sleep quality might modulate amygdalar functional connectivity and not vice versa (79). Additionally, patients with narcolepsy have lower GM volume in the amygdala relative to controls, suggesting a possible unidirectional relationship between sleep quality and amygdala volume (80).

Our finding of sex-specific differences in cerebellar volume among cannabis users was not present in other regions with high CB1-R density, such as the amygdala and the hippocampus. Preclinical studies in rats have shown that chronic THC caused downregulation and desensitization of CB1-R in cerebellum, and these decreases were especially large in females (9). Other studies have reported higher baseline CB1-R density in female compared to male rats, although the cerebellum was not examined (10). Human PET studies have similarly found that females have higher baseline CB1-R availability than males in many brain regions (81), including cerebellar cortex (82). Given that CB1-R density was influenced by the estrous cycle in preclinical studies, it is possible that female sex hormones play a role in sex differences in CB1-R availability as well as sex differences on cannabis effects in brain and behavior (10). Animal models could be used to test if sex differences in CB1-R density prior to and after chronic THC exposures may confer female vulnerability to potential neurotoxic effects of cannabis on cerebellar structure and function. Additionally, while initial human PET studies found that CB1-Rs in both sexes were downregulated in response to chronic cannabis use (69), future longitudinal studies should examine whether there are sex differences on the association of CB1-R downregulation with the severity of CUD. This is especially important given that there are currently no FDA-approved pharmacological treatments for CUD, and that one promising candidate, the fatty-acid amide hydrolase (FAAH) inhibitor PF-04457845, was recently shown to reduce cannabis withdrawal severity and promote abstinence, but only men with CUD were included in the trial (83). Thus, much work remains to be done to see if treatments show similar improvements in females and if they do so in part via cerebellar mechanisms. Our group has previously proposed that downregulation of CB1R in subjects with cannabis dependence might increase vulnerability to cortical thinning, suggesting that CB1R availability can lead to structural changes in the brain (21). Another study found that some heavy cannabis users have a genetic predisposition toward cannabis dependence due to a functional single nucleotide polymorphism affecting cannabis receptor-1 gene expression; among cannabis users, minor relative to major allele carriers had lower volume in the nearby hippocampus, but not the amygdala (84). However, cerebellum volume was not examined in this study, and it is possible that there is a similar connection between CB1R and cerebellar volume. Future studies should examine this possibility to uncover the link between CB1R activation/availability and amygdalar/cerebellar volume.

We also observed that females' self-reported sleep quality (as indexed by the global PSQI score) was similarly more vulnerable to the negative effects of cannabis use than for males. Given that patients with degenerative diseases of the cerebellum such as cerebellar ataxia commonly report sleep disturbances, poor subjective sleep quality, restless leg syndrome, and REM behavior disorder, it is plausible that the cerebellar volume loss in female cannabis users contributed to their poor sleep quality (85, 86). However, in our study the effects of cannabis on cerebellar volumes did not mediate the effects of cannabis on sleep quality, which is likely to reflect a more complex association between cannabis effects in brain structure and function. Similarly, the effects of cannabis on sleep quality did not mediate its effects on brain volume, which might also indicate distinct neurobiological processes underlying these two effects. Given the observational nature of this study, we are unable to rule out the possibility that women to start with had lower sleep quality than men as has been reported by other studies (25–28), though in our current study sleep scores in control males did not significantly differ from those in control females. It is also possible that a mismatch between expectation and reality in how cannabis helps with sleep may play a role in self reports; in a majority female (67%) sample of cannabis users, while both frequency and presence of cannabis use were associated with the expectation of improved sleep, cannabis use was actually associated with poorer subjective sleep quality (87). Finally, we observed that females who reported first using cannabis in early adolescence tended to report the worst sleep quality, which aligns with a recent large-scale twin study (*n* = 1,656) that reported that regular cannabis use at a young age correlated with shorter sleep duration in adulthood (88). Given the differences in socialization, development, and expectations associated with cannabis use, women may be more vulnerable to the negative sleep effects of cannabis abuse at younger ages than men. These data complement a large body of literature suggesting that early-onset cannabis use is strongly associated with poor neuropsychiatric outcomes (89), and again highlight sex differences as an important future avenue of investigation.

### Limitations

The HCP provides a large, high-quality dataset of MRI-based and behavioral data (90). Nonetheless, given that scans were completed between 2012 and 2015 by the WU-Minn Consortium, in Missouri and Minnesota, where medical marijuana was not legalized until 2014 (albeit restrictively and only for certain chronic conditions) it is likely that most participants used cannabis recreationally, not as prescribed by a doctor. While this allows us to compare a uniform population of chronic recreational users to non-users, we were unable to investigate any effects of medical cannabis use on sleep quality or cerebellar volumes. We also do not have any information on whether participants were using cannabis to self-treat sleep issues. It is possible that when used for medicinal purposes and with low-THC strains that are less likely to lead to CUD (91), cannabis may not have a negative impact on sleep (15). Additionally, while we matched the chronic cannabis use group with controls on several important demographic variables including a composite score reflecting current and past alcohol consumption, cannabis users did not match controls on measures of tobacco usage. Considering that females had lower nicotine use than males and yet they showed greater effects than males, and that we covaried for tobacco use, it is likely that the effects on sleep and cerebellar volumes reflect cannabis and not nicotine effects. Nonetheless we cannot completely rule out that the interaction between cannabis and tobacco use contributed to the effects in brain and sleep quality. Finally, this analysis is limited by the imbalance in the number of male and female subjects in our sample: each one of the groups had twice as many males as females. This sex imbalance is representative of the U.S. population at large, since the majority of people who use cannabis are male (92–98), though this imbalance may affect our results on group differences between cannabis users and controls (as noted in the discussion) and limit our statistical power. This limitation emphasizes the need to include equal numbers of men and women in clinical studies, so that sex differences can be rigorously examined.

### Future Directions

Future studies should include polysomnography measurements or other objective measures of sleep architecture and duration in addition to self-reported sleep data. The impact of cannabis use on sleep requires further exploration, for a recent meta-analysis reported that most of the prior studies reported sleep as a secondary outcome and were done on small sample sizes using unvalidated measures (99). Further, studies on the effects of cannabis on sleep architecture and its response to treatment are sorely needed. Finally, future studies should attempt to account for THC potency and a richer quantification of doses and frequency of cannabis use (100), to discern the effects of light vs. heavy cannabis use in general and in the context of these sex-dependent effects on sleep and cerebellar volume.

## Data Availability Statement

The original contributions presented in the study are included in the article/supplementary material, further inquiries can be directed to the corresponding author/s.

## Ethics Statement

The studies involving human participants were reviewed and approved by Washington University in St Louis IRB. The patients/participants provided their written informed consent to participate in this study.

## Author Contributions

KM, PM, G-JW, and NV: study conception. KM, PM, and DT: data analysis. KM: first draft of manuscript. All authors: editing.

## Conflict of Interest

The authors declare that the research was conducted in the absence of any commercial or financial relationships that could be construed as a potential conflict of interest.
